# Dietary Intake, Mediterranean Diet Adherence and Caloric Intake in Huntington’s Disease: A Review

**DOI:** 10.3390/nu12102946

**Published:** 2020-09-25

**Authors:** Christiana C. Christodoulou, Christiana A. Demetriou, Eleni Zamba-Papanicolaou

**Affiliations:** 1 Neurology Clinic D and Bioinformatics Department, The Cyprus Institute of Neurology and Genetics, The Cyprus School of Molecular Medicine, Nicosia 2371, Cyprus; christianachr@cing.ac.cy; 2 Bioinformatics Department, The Cyprus Institute of Neurology and Genetics, The Cyprus School of Molecular Medicine, Nicosia 2371, Cyprus; 3 Neurology Clinic D, The Cyprus Institute of Neurology and Genetics, The Cyprus School of Molecular Medicine, Nicosia 2371, Cyprus; 4 Department of Primary Care and Population Health, University of Nicosia Medical School, Nicosia 2371, Cyprus; demetriou.chri@unic.ac.cy

**Keywords:** Huntington’s disease, Mediterranean Diet adherence, macronutrients, micronutrients, caloric intake

## Abstract

Decades of research and experimental studies have investigated Huntington’s disease (HD), a rare neurodegenerative disease. Similarly, several studies have investigated whether high/moderate adherence to the Mediterranean Diet and specific macro and micronutrients can decrease cognitive loss and provide a neuroprotective function to neurons. This review systematically identifies and examines studies that have investigated Mediterranean Diet adherence, micro- and macronutrients, supplementation and caloric intake in people with HD, in order to identify if dietary exposures resulted in improvement of disease symptoms, a delay in age of onset or if they contributed to an earlier age of onset in people with HD. A systematic search of PubMed, Directory of open access journal and HubMed was performed independently by two reviewers using specific search terms criteria for studies. The identified abstracts were screened and the studies were included in the review if they satisfied predetermined inclusion criteria. Reference screening of included studies was also performed. A total of 18 studies were included in the review. A few studies found that patients who had high/moderate adherence to Mediterranean Diet showed a slight improvement in their Unified Huntington’s Disease Rating Scale and Total Functional Capacity. In addition, people with HD who had high Mediterranean Diet adherence showed an improvement in both cognitive and motor scores and had a better quality of life compared to patients who had low Mediterranean Diet adherence. Furthermore, a few studies showed that supplementation with specific nutrients, such as triheaptanoin, L-acetyl-carnitine and creatine, had no beneficial effect on the patients’ Unified Huntington’s Disease Rating Scale score. A few studies suggest that the Mediterranean Diet may confer a motor and cognitive benefit to people with HD. Unfortunately, there was little consistency among study findings. It is important for more research to be conducted to have a better understanding of which dietary exposures are beneficial and may result delaying age of onset or disease progression in people with HD.

## 1. Introduction

### 1.1. Huntington’s Disease and HD Genetics

Huntington’s disease (HD) is a rare, inherited autosomal dominant progressive neurodegenerative disease affecting the medium spiny neurons [1] of the basal ganglia of the central nervous system (CNS) [2]. Neuronal degeneration mainly occurs in the striatum of the basal ganglia and cortex [3]. Clinical features include movement impairment such as chorea, an involuntary twitching movement and incoordination, cognitive decline and behavioral impairments such as depression, personality changes and psychosis. As the disease progresses, all symptoms worsen and the involuntary movements become more prominent [4]. The Unified Huntington’s Disease Rating Scale (UHDRS) is used to evaluate motor, cognitive, behavioral and functional domains in HD [5]. HD usually has a mean age of onset of approximately 40 years and the average life expectancy of people with HD is 17 years after the onset of symptoms [4]. The huntingtin (HTT) gene, which is mutated in HD, consists of cytosine-adenine-guanine (CAG) which are repeated multiple times. This is known as a CAG trinucleotide repeat [6]. When the CAG repeat length reaches a certain threshold it produces an altered form of the HTT protein, called mutant huntingtin protein (mHTT). The CAG trinucleotide is repeated between 10–35 times in healthy individuals. Individuals that have between 36–39 CAG repeats may or may not develop the signs and symptoms of the disease, meaning that there is reduced penetrance. However, individuals with 40 or more repeats will always develop the signs and symptoms of HD. The trinucleotide repeat varies in length between individuals and generations and it is the main predictor for age of onset, disease severity and occurrence of HD. However, despite the number of CAG repeats being the major determinant of age of onset, there is still variation in age of onset of the disease among individuals, which remains unexplained [2].

### 1.2. Micronutrients, Macronutrients and Neurodegenerative Diseases

Over the last century, remarkable progress has been made in the field of human nutrition both in the identification of essential minerals and vitamins, amino acids and fatty acids and in the understanding of their role in metabolism and disease prevention. Progress has also been made in the identification and description of metabolic pathways and the classification of genetic variants that may affect metabolism [7]. Humans consume meals that contain complex combinations of macronutrients (carbohydrates, fats and proteins) and micronutrients (vitamins and minerals), and various food items may play a role in preventing or delaying the onset of diseases [7].

Micronutrients such as vitamin D, E, and C are essential elements needed in small amounts, which are necessary to maintain physiological function, health, development and growth of the organism [8]. Previous studies have shown the importance of micronutrients such as vitamin E, vitamin C, carotenoids and flavonoids as exogenous antioxidants that are able to eliminate free radicals, that can contribute to neurodegeneration [9]. A randomized controlled trial where 4447 French participants received daily vitamin C, β-carotene, vitamin E, selenium and zinc supplementation or a placebo, the study demonstrated that participants who had received a combination of antioxidant supplementation, had better episodic memory scores six years after the trial was completed [9].

Macronutrients such as carbohydrates, fats and proteins are a class of chemical compounds consumed by humans in large amounts, and are required for the maintenance of normal human body functions, including neuronal health. For example, fatty acids were shown to modulate the risk of cognitive impairment and dementia [10]. However, excessive dietary intake of some of these macronutrients may lead to health issues. A study in rodents with Parkinson’s Disease (PD), demonstrated that diets high in fat, worsened the progression of PD by demonstrating increased dopamine (DA) depletion in the striatum, substantia nigra and nigrostriatal pathway [11].

Overall, various studies have shown that chronic diseases including neurodegenerative diseases may be prevented or delayed by adapting to a healthier diet and lifestyle [7].

### 1.3. Mediterranean Diet and Neurodegenerative Diseases

Perhaps the most well researched dietary pattern is the Mediterranean Diet (MD). There is a large amount of research data available, which demonstrates the health benefits of the MD and its effect on lowering the incidences of chronic diseases such as cardiovascular disease, metabolic syndromes, breast cancer and on improving longevity [12]. When investigated as a dietary pattern, previous studies have shown the beneficial effect of the MD also in neurodegenerative diseases. Results indicate that individuals who adhere to the MD have a decreased occurrence of dementia and AD [13]. The MD also has beneficial effects in reducing cognitive decline. A cohort study of 1393 patients who had normal cognitive function at baseline reported that adherence to the MD was related to a decrease in the risk of experiencing cognitive decline after 4.5 years of follow-up [13,14].

Furthermore, a population study was conducted in Chicago and involved 4000 participants aged >65 years for a period of 8 years. The results demonstrated an inverse association between MD adherence score and cognitive decline [13,15]. Lastly, studies have shown that a higher adherence to the MD may delay the onset of AD and may be associated with late age of onset of PD [16].

The MD consists of a high consumption of plant based foods (fruits, vegetables, nuts and legumes), fish and extra virgin olive oil which is main source of monounsaturated fat (MUFA) and low to moderate intake of wine and low intake of red meat, poultry and dairy products [16]. Both analytical and experimental studies have shown a relationship between increased consumption of food categories that are abundant in the MD and reduction in neurodegenerative disease risk [13]. On the other hand, an increase in the intake of calories, meats and fats (the latter two being sparse in the MD) are associated with a greater risk for disease development [13]. Overall, the MD provides nutrients, phenolic compounds and antioxidants that protect the nervous system from neurodegeneration; therefore, it may reduce the risk and/or progression of devastating neurodegenerative diseases [13,17]. An appropriate diet and nutritional intake is vital for the maintenance of good health and functionality. Furthermore, it is an effective way to decrease the burden of several health conditions, such as obesity and diabetes [17].

Despite the wealth of evidence demonstrating a protective role of the MD and several micro- and macronutrients in neurodegeneration, the effects of diet on HD have not been extensively studied. As certain diet components are increasingly highlighted as neuroprotective, a detailed investigation of their role in HD onset and clinical characteristics is warranted. However, this investigation needs to be preceded by a review of all relevant studies published to date to streamline any existing results thus highlighting research priorities. The aim of this study, attempts to summarize studies, evaluating the effects of macronutrients and micronutrients, calories of HD, MD adherence and dietary intake in HD.

## 2. Materials and Methods

### 2.1. Search Strategy and Study Selection

**Figure 1:**
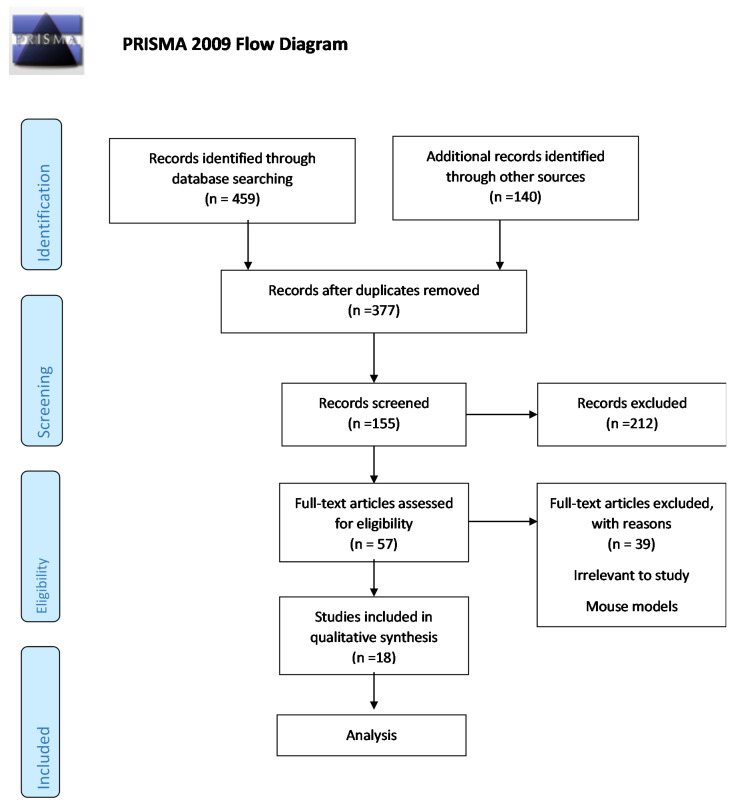
PRISMA Flow Diagram. The following flow diagram indicates the identification, screening, eligibility and studies included in this review and also studies and the reasons for their exclusion from the study.

In order to identify studies that investigate any association between diet (MD adherence, or food group consumption or nutrient intakes) with HD in human subjects, a literature review was conducted using the electronic databases of PUBMED, Directory of open access journal and HubMed for studies published until November 2019. The following combination of search terms was used: “Huntington’s Disease AND diet”, “Huntington’s Disease AND dietary components”, “Huntington’s AND disease AND diet OR dietary components OR dietary patterns”. No protocol exists for this review. A total of 599 articles were retrieved from the search, 222 of which were duplicates. The abstracts were screened independently by two investigators and, if considered relevant, full articles were subsequently reviewed. Data was extracted from the identified studies, independently by two investigators, using a common data-extraction form. Studies involving animal models, non-English or irrelevant studies were excluded (Figure 1). The cited references of the identified studies were also searched for additional relevant publications. The article selection process is outlined in Figure 1. The PRISMA checklist is illustrated in (Table S1).

The following types of studies, were included in review (1) randomized controlled trials, (2) non-randomized intervention trials, (3) case-control studies and (4) cohort studies. The studies, that were included in our review were studies investigating (1) dietary intake and patterns, (2) MD adherence, (3) nutritional supplementation, and (4) caloric intake, in humans. Similar studies in HD mouse models were excluded. Furthermore, the studies, that investigated and assessed MD adherence such as Rivadeneyra et al., 2016 and Marder et al., 2013, used the Trichopoulou score with a range of 0–9 to assess MD adherence [16,18,19].

### 2.2. Quality Analysis of Studies

A quality of analysis was conducted for the studies included in our review. The Newcastle-Ottawa scale (NOS) was used to assess the quality of case-control and cohort studies [20] and the Cochrane risk of bias tool (https://www.cochrane.org/) was used to assess the quality of randomized controlled studies. The NOS assigns a maximum of nine points for the three risk of bias domains: (1) selection of study groups (four points); (2) comparability of groups (two points); and (3) ascertainment of exposure and outcomes (three points) for case–control and cohort studies. The NOS questions are slightly different between case-control and cohort studies. The questions for each study type can be seen in (Figures S1 and S2) respectively. Additionally, the analysis of quality of analysis of case-control and cohort studies using NOS are illusrated in (Tables S2 and S3) respectively for NOS.

The Cochrane risk of bias tool, assesses risk of bias and the quality of studies in randomized controlled studies, by asseesing the following domains (i) slection bias: random sequence generation, allocation concealmet, (ii) reporting bias: selective reporting and (iii) other bias: other sources of bias, (iv) performance bias: blinding (participants and personnel), (v) detection bias: blinding (outcome assessment), (vi) attrition bias: incomplete outcome data. The Cochrane domains covered for randomized controlled trials are illustrated in (Figure S3). The quality of analysis of randomized controlled trials is illustrated in (Table S4).

## 3. Results

An overview of the studies included in the review, is illustrated in (Table 1) and includes, (i) study country, (ii) range of ages, (iii) range of participants and (iv) study type. In total 18 unique, relevant studies were included in this review, which are presented in (Table 2) As outlined below, most of the included studies supported the hypothesis, that specific dietary intakes can delay disease progression and improve the motor score of people with HD, as measured using the UHDRS score. On the other hand, some studies have found that consumption of certain dietary components may lead to early age of onset in HD. Additional information for each study can be found in the supplementary section.

### 3.1. Supplementation

Different dietary supplementations such as ethyl-EPA [21,22], trihepatonin [23], L-acetyl-carnitine (LACC) [24], carnitine [25], creatine [26], uric acid (UA) [27] nutritional supplementation [28] and oral supplementation [29] were investigated to identify if there was an improvement or no improvement in the motor function of people with HD. There was no improvement in the motor function of the intent to treat (ITT) cohort of patients for ethyl-EPA [21]. However, in the protocol violations (PP) cohort, the ethyl-EPA group showed improvement in their motor function when compared to placebo. Another study with ethyl-EPA supplementation [22] showed that ethyl-EPA was effective in significantly decreasing global cerebral atrophy during the first months of treatment. More specifically, a reduction in the caudate and thalamus regions was seen in the ethyl-EPA treated group. Ethyl-EPA appears to exhibit some beneficial effect on motor function and decreasing brain atrophy. Triheaptanoin [23] showed no significant improvement in the UHDRS scores of patients before and after treatment.

Similarly, no association was found between LACC treatment [24] and improvement in cognition, abnormal involuntary movement scale (AIMS) and verbal fluency. The only association identified was between LACC and reaction time. No symptom improvement or delayed disease progression was seen in patients with the use of LACC.

Furthermore, no association was identified between creatine therapy and improvement in motor or cognitive function of people with HD. In addition, long-term creatine supplementation did not show any significant improvement in TMS, functional capacity and neuropsychology testing [26].

Patients with hypocarnitinenmia were treated with carnitine supplementation and patients without hypocarnitinenmia were not treated with carnitine supplementation. No difference was found between UHDRS and mini mental state examination (MMSE) scores between the two groups. However, during the first months of carnitine supplementation there was an improvement in UHDRS scores and a decrease in MMSE scores in the treatment group. During the last months of the study no significant association was observed with improvement in UHDRS scores, MMSE decline, falls and violent episodes between the two groups [25]. An additional study investigated the use of oral nutritional supplements in people with HD. [24]. Dietary assessment of macronutrients, energy intake, and total energy intake were assessed in people with HD along with UHDRS scores. No change was observed in HD patients UHDRS scores from day 0 to day 90, indicating no association between diet and UHDRS scores [28]. The relationship between uric acid (UA) and progression of HD was investigated by looking at the functional decline in people with HD [27]. An association was found between baseline UA and change in the TFC over a 30-month period from the lowest to highest quintile. More specifically, increasing UA levels were associated with less worsening in the total motor scores from the lowest to highest quintile. This suggests an association between baseline UA concentration and slower progression of HD.

Supplementation was also studied Cubo et al., 2015 [29]. The study found that high intake of water-soluble vitamins and minerals was more common in advanced HD. No significant benefit was observed with supplement intake, in motor and cognitive impairment or in the functional state of patients.

### 3.2. Micronutrients and Macronutrients

Some studies investigated the nutritional status of people with HD to determine whether micronutrient and macronutrient intake helped in improving disease symptoms or delaying disease progression [18,30,31,32].

Macronutrients and energy intake were compared between an HD group and controls [30], and only energy intake and high consumption of carbohydrates were increased in the HD group. The high-energy intake is likely to be associated with the higher consumption of carbohydrates. The increase in the UHDRS subscale was not significant to identify an association.

Nutritional and caloric intake was investigated between HD patients vs. HD descendants vs. controls, but no association was identified between the groups [32]. However, it was observed that choreic patients had a significantly higher intake of calories and micronutrients. Also, HD women were more likely to have an iron deficient diet, and vitamin C and Niacin, were found to be deficient in all choriec patients. Despite the above, no nutrient intake was associated with HD clinical outcomes.

The effects of a high protein diet (HPD) in HD were investigated between HD patients and controls [31]. The HPD showed a slight increase in citrulline but ammonia levels were not affected by HPD. Citrulline levels were higher in HD patients compared to controls. During the follow-up study, there no significant change in citrulline concentration in people with HD. The UHDRS and MMSE scores of people with HD were accessed from baseline to 24 months, and there was no significant association between citrulline and UHDRS scores, independence score (IS) and functional capacity. At 12 months of follow-up, there was a negative association between citrulline levels and disease duration. Although there was no significant association between citrulline concentration, an alteration in motor function and functional capacity was seen at 18 and 24 months of follow-up. The association between citrulline concentration and disease association might suggest that the nutritional status of HD patients, specifically in the end stage of the disease, might influence their citrulline levels. High caloric intake was seen in severe HD compared to mild-moderate HD patients [31]. Caloric intake and body mass index (BMI) were similar when compared between pre-manifesting and manifested HD patients. Higher intake of fat and micronutrients was seen in the severe HD group compared to the mild moderate groups. Dietary intake in this study was not associated with improvement in functional state of patients.

### 3.3. MD Adherence

Little is known on how the importance of the MD and dietary intake may help in delaying disease progression and providing neuroprotection to neurons. However, a number of studies [31,32] investigated the effect of MD adherence and dietary intake in delaying HD progression or improving the UHDRS scores and phenoconversion in patients. Others [33] investigated certain dietary factors and their effect on HD progression and age of onset (AAO) while others [34] investigated coffee consumption and AAO.

Rivadeneyra et al., 2016 [18] found that, HD patients who had high or moderate MD adherence, had higher intake of macronutrients but lower consumption of dairy products and higher intake of micronutrients compared to low MD adherence. Moderate/high MD adherence was characterized with a higher intake of MUFA/SFA and polyunsaturated (PUFA) + MUFA/saturated fatty acids (SFA) which was associated with a slight improvement of TFC and UHDRS cognitive scores compared to low MD adherence. Also, moderate to high MD adherence, was associated with a slight improvement in the UHDRS motor and cognitive scores of patients. However, HD severity was similar between subjects of low vs. moderate/high MD adherence.

Phenoconversion was investigated in terms of the relationship between BMI, MD adherence, caloric intake and specific food group intake [32]. Higher BMI was associated with lower MD adherence, but BMI was not identified as a risk factor for phenoconversion. Similarly, MD adherence was not identified as a predictive factor for phenoconversion. However, higher caloric intake was found to be significantly positively associated with phenoconversion. Looking at particular food groups, high consumption of dairy products was significantly associated with a two-fold increased risk of phenoconversion.

### 3.4. Lifestyle and Dietary Patterns

Lifestyle factors and dietary factors may influence both the age of onset and rate of progression in HD. One study [33] identified no association between the dietary intake of alcohol, coffee, fruit juice, tea, cheese and fish and HD age of onset. However, a significant association was identified between dairy consumption and more specifically milk and early age of onset. Milk and dairy product consumption was negatively correlated with age of onset.

Simonin et al., 2013 [34] investigated, caffeine consumption and age of onset or functional and motor decline. There was a significant association between average caffeine intake before and after disease onset. Furthermore, increased caffeine consumption of ≥190 mg/day before disease onset was associated with an early AAO. However, there was no association between UHDRS scores and caffeine consumption. Therefore, increased caffeine intake was negatively associated with AAO but not with symptoms.

### 3.5. Caloric Intake

A study by Marder et al., 2009 [35] found no difference in overall macronutrient intake between CAG ≥ 37 and CAG < 37 groups. However, carbohydrate intake was higher in the CAG ≥ 37 group. In the same study, caloric intake was significantly associated with CAG repeat length and with the estimated 5-year probability for HD onset in the expanded CAG ≥ 37 group. The association between caloric intake and the 5-year probability of disease onset is likely due to a higher consumption of calories in individuals during the pre-manifesting phase of HD, perhaps in an attempt to maintain their energy balance and weight.

Mochel et al., 2007 [36], showed that HD patients had significant weight loss compared to controls. HD men had lower BMI compared to controls and total caloric intake was inversely associated with weight and lean body mass, indicating that people with HD show an early hypermetabolic state. Weight loss was also observed in pre-symptomatic carriers even though they had higher caloric intake compared to controls. The early weight loss seen in HD may be associated with a systemic metabolic defect.

## 4. Discussion

Over the last decade, a number of studies indicated the importance of a healthy lifestyle, dietary intake and diet in delaying or providing protection against the occurrence of chronic diseases [13]. Studies have assessed the associations between food groups, nutrients and diseases, and there is a general agreement about the role of nutrients and diet in the etiology of commonly caused diseases such as cardiovascular disease. Experimental data has looked at the relationship between increased consumption of fruits, vegetables and a decreased risk in chronic diseases, whereas high consumption of meats, fats and increase in total caloric intake and body weight result in a greater risk of developing disease [13,17]. Studies assessing diet, nutritional status and dietary intake have also been undertaken in neurodegenerative diseases such as AD, PD, ALS and HD [13,17].

However, there is a lack of studies investigating the effect of dietary intakes on HD onset and/or progression and on symptom improvement. The purpose of this review was to identify if certain nutrients, dietary habits or dietary patterns such as the MD can delay the onset and/or progression of HD and if it has a beneficial effect on HD symptoms.

The potential mode of actions of the supplements and how they play a role in HD, are discussed below. The Ethyl-EPA is a semi-synthetic compound, it a derivative of EPA which is a polyunsaturated, omega-3 fatty acid [21,22]. Previous studies have identified. Ethyl-EPA is able to target mitochondrial function by acting on the peroxisome proliferator activated receptors [21]. Additionally, Ethyl-EPA has demonstrated to be effective in preventing neuronal loss, decreasing mitochondrial dysfunction by decreasing the activity of the JNK pathway and inhibition of caspase activation for apoptosis [21]. JNK is part of the mitogen-activated protein kinase family and they are responsive to stress stimuli such as osmotic and heat shock, ultraviolet irradiation and cytokines. JNKs also play a role in T-cell differentiation and in apoptosis [21].

LACC is the shortest form of acylcarnitine and LACC is naturally found in the body, it formed in cells by the enzymatic addition of an acetyl group to carnitine, it plays a role in brain metabolism, it is likely to be transported across the blood-brain barrier (BBB) [37]. LACC has been identified to play a role in cholinergic neuronal transmission and may also play a role in increasing gamma-aminobutyric acid (GABA) concentration in the brain [24,37]. A previous study investigated mitochondrial modulators in HD, and how they may reverse mitochondrial dysfunction and cognitive decline [37]. The study had administrated 3-Nitropropionic acid (3-NP) to mice, this results in comprised mitochondrial functions such as (i) impaired activity of mitochondrial respiratory chain enzymes, altered cytochrome levels and a decrease in mRNA expression of respiratory chain complexes; (ii) enhanced mitochondrial oxidative stress indicated by increased malondialdehyde, reactive oxygen species and nitrite levels, and also a decrease Mn-superoxide dismutase and catalase activity; (iii) mitochondrial structural, such as mitochondrial swelling, reduced mitochondrial membrane potential, (iv) increased cytosolic cytochrome c levels, caspase-3 and -9 activity and altered expression of apoptotic proteins (AIF, Bad, and Bax); and (v) impaired cognitive function [38]. A combination of mitochondrial modulators such as alpha-lipoic acid and acetyl-L-carnitine, ameliorated 3-NP induced mitochondrial dysfunction, oxidative stress, mitochondrial structural changes and behavioral deficits, suggesting their therapeutic efficacy in HD management [38].

Triheptanoin is a triglyceride and composed of seven carbons, it has been used in clinical trials to treat patients with long chain fatty acid oxidation disorders. Patients treated with triheptanoin are less likely to develop hypoglycemia, cardiomyopathy and hepatomegaly [39]. Triheptanoin is able to refill the pools of intermediates of the citric acid cycle. The citric acid cycle, consists of a series of chemical reactions, that is used by aerobic organisms to release stored energy via the oxidation of acetyl-CoA derived from carbohydrates, fats, and proteins [23], by increasing citric acid cycle intermediates in HD, the energy deficits observed in people with HD can be reduced [23].

Carnitine is a quaternary ammonium compound and it is involved in the metabolism of mammals, plants and bacteria. Carnitine, is found in a number of food sources such as beef steak, ground beef, codfish, chicken and cheese and various other foods [39]. It is also an important regulator of lipid metabolism. Carnitine acts a lipid transporter, it transports long chain fatty acids into the mitochondrial matrix, where beta-oxidation occurs, here the lipids are oxidized for energy production via the citric acid cycle [25]. Carnitine is concentrated in skeletal and cardiac muscle that metabolize fatty acids as an energy source [25]. However, there is no clear indication of how carnitine may delay age of onset and disease symptoms as no significant difference was identified for UHDRS scores, MMSE decline, falls and violent episodes in people with HD [25].

Creatine is amino acid that is primarily located in the muscles and brain and it can be obtained from the foods, such as seafood and red meat. Additionally, it can also be synthesized synthetically. A randomized controlled clinical trial study using creatine was conducted for HD. 40g of creatine monohydrate was given daily to patients with HD who are stage I or stage II for a period of 48 months. The TFC between baseline and end of follow-up was measured and additional measures such as clinical scores, tolerability, and quality of life. Safety was assessed by adverse events and laboratory studies. However, this study did not identify a beneficial effect of creatine on HD. Additionally, the study failed to observe the improvement in TFC and improvement in other scores such as clinical scores, tolerability and quality of life [40]. Further research is required to assess the beneficial effects of creatine in HD.

UA has antioxidant properties; it is scavenger of oxygen radicals and oxidative damage, an increase in UA concentration may have a therapeutic and protective role against oxidative damage that is associated with neurodegenerative diseases such as HD [27]. It was identified that UA has neuroprotective properties, by decreasing brain injury following stroke, by reducing neuronal cell death and oxidative damage [27]. Increase UA concentration may lower the risk of developing neurodegenerative disease such as PD, AD and HD [27].

Overall, the results of this review suggest an improvement in the cognitive and motor scores and a better quality of life in people with HD who had high MD adherence. Also, certain food groups such as a higher consumption of milk and dairy products and caffeine consumption greater 190mg/day seem to be associated with an earlier age of onset.

We attempted to perform a comparison between additional dietary patterns such as the ketogenic, MIND and DASH diets and HD. However, we identified no studies for the various dietary patterns and HD, in people with HD. Further research is required to compare the various dietary patterns with MD and their effect on HD.

The studies included in this review are not without their limitations, such as small sample size, short duration of study, no correction of missing data, and possible misclassification of the dietary exposures. Perhaps the most significant limitation of included studies is that most studies were cross-sectional (liable to the problem of reverse causation), therefore no conclusions can be reached regarding the temporality of associations between diet and clinical phenotypes. Only a few studies involved follow-up of participants which allows for more confidence in inferring whether diet affects clinical phenotypes.

Secondly, self-reporting of dietary exposures, which was often, used can lead to exposure misclassification, since the participants may not respond truthfully either because they are unable to recall their dietary intake and habits or they would like to present themselves in a socially acceptable manner [27,31,32,33,34,35].

Furthermore, some studies administered a 3-day dietary record, which can capture some of the day to day variation, but some participants did not habitually record their dietary intake over 3 days [24,26,27,31,35]. In some cases, 24-h recalls were administered but this approach is unable to describe the usual dietary intake of individuals [26,29,36]. Furthermore, there was no validation of the FFQs used, and this can affect the quality of the data and the results obtained may be less trustworthy than with a validated questionnaire [24,28,29,35]. Validity is important, as it helps to collect better quality data and there is greater credibility in the data.

Another limitation that was common to many studies was that the study duration and/or follow-up period was too short to enable detection of meaningful changes in HD symptoms and/or progression [16,18,19,21,22,27,30]. The small sample study was a limitation to many studies [16,19,21,22,23,24,28,29,30,31,33,34,35] which coupled with the analysis of several exposures in some studies [22,24,25,27,28,31,32,34,35], this limits the statistical power to detect any associations.

Other limitations of the included studies, include the lack of correction for missing data [26,31], the failure of the blood samples collected to analyze all covariates of interest [32], and the non-standardized handling of serum samples which may affect the serum concentration of the analyte of interested and this can affect the outcome [22].

Lastly, the inability to control for confounders that were not investigated in the study, the inclusion of study participants with an early HD onset (who may display a more severe phenotype) and the question of the representativeness of the results with respect to the rest of the HD population [25], were limitations less common in the included studies but are worthy of mention after all.

## 5. Conclusions

The results of this review suggest an improvement in the cognitive and motor scores and a better quality of life in people with HD who had high MD adherence. Moreover, a high caloric intake was repeatedly observed in people with HD but it is likely due to the higher consumption of calories in order to maintain their weight in the pre-manifesting stage of HD. Furthermore, the results suggest that certain food groups such as a higher consumption of milk and dairy products and caffeine consumption greater 190mg/day are associated with an earlier age of onset.

Although, higher intake of dairy and milk products and caffeine were to found to play a role in HD, causation relationships and lifestyle advice cannot be inferred from these mostly cross-sectional studies and more longitudinal studies are needed to determine whether the above mentioned food groups along with others contribute to HD pathology. Furthermore, new studies in the field need to be conducted with a larger number of participants; longer study periods and validated FFQs in order to identify and determine if the consumption of certain foods contributes to HD and to the earlier age of onset. Good quality evidence from better designed studies can offer invaluable research insights into the relationship between diet and HD onset and progression and can be used in both non-pharmacological and pharmacological interventions to modify onset and/or progression of HD. 

## Figures and Tables

**Table 1:** Overview of studies included in the review.

Study Reference	Study Countries	Range of Participants	Range of Ages	Study Type
**Marder et al., 2013 [16]**	**United States of America (USA)**	People with HD recruited from Prospective Huntington at Risk Observational study (PHAROS) 41 HD group sites in US and Canada 1001 participants enrolled in PHAROS 738 individuals completed NIH FFQ Inclusion of 211 HD patients with expanded CAG ≥ 37	25–57 years	Prospective cohort study
**Rivadeneyra et al., 2016 [18]**	**Spain**	98 Spanish people with HD and pre-manifesting HD carriers of the European Huntington Disease Networks	48 (range 38–60)	Cohort study
**Puri et al., 2005 [19]**	**United Kingdom (UK)**	135 Symptomatic people with HD 67 in Ethyl EPA 68 in placebo group	50 ± 9.3 (Ethyl EPA group) 49 ± 9.0 (Placebo group)	Double blind, randomized controlled trial (RCT)
**Puri et al.,** **2008 [21]**	**United Kingdom (UK)**	34 Symptomatic people with HD 6 in Ethyl-EPA group 18 in placebo group	51.3 ± 2.5 48.7 ± 2.2	Double blind, randomized controlled trial (RCT)
**Mochel et al.,** **2010 [22]**	**France**	6 people with HD with abnormal CAG repeat expansions 5 females 1 male	NR	Short term cohort clinical trial study
**Goetz et al.,** **1990 [23]**	**United State of America (USA)**	10 HD patients in a double blind RCT, crossover design 4 men and 6 women with HD	51.6 (16.7)	Double blind placebo controlled cross-over study
**Cuturic et al.,** **2013 [24]**	**United State of America (USA)**	23 institutionalized people with HD 6 people with HD treated with hypocarnitinemia 17 people with HD without hypocarnitinemia	43.4 (10.3) 51. 9 (7.9)	Retrospective study
**Tabrizi et al.,** **2003 [25]**	**United Kingdom (UK)**	10 people with HD and 3 HD mutation carriers 4 age matched controls	NR	Open-label pilot study
**Auigner et al.,** **2010 [26]**	**United State of America (USA)**	347 early HD patients	18–75 (47.9 years)	Double blind, randomized controlled trial (RCT)
**Trejo et al.,** **2005 [27]**	**Mexico**	30 people with HD 70% male 30% female	46 (range 20–69)	Interventional study cohort study
**Cubo et al.,** **2015 [28]**	**Spain**	224 Spanish people with HD patients and carriers from the European HD registry (EHDN) Control group from Spanish population obtained from EHIDE	47.41 ± 14.26	Observational, cross-sectional study
**Trejo et al.,** **2004 [29]**	**Mexico**	25 people with HD 25 age and sex matched controls	46 ± 12 (21–70) 46 ± 8 (23–72)	Case-control study
**Chen et al.,** **2015 [30]**	**Taiwan**	30 people with HD 19 men and 11 women 23 controls 12 men and 11 women 14 HD out of 30 people with HD (2 year pilot study) 22 HD patients (1 year follow up)	44.7 ± 11.4 42.26 ± 2.6	Intervention and cohort study
**Morales et al.,** **1989 [31]**	**Venezuela**	18 choreic patients 31 offspring (1st generation) 19 individuals (2nd generation) 7 individuals (3rd generation) 40 controls	15 to 57 years 12 to 72 years 14 to 45 years 18 to 32 years 14 to 72 years	Case-Control study
**Buruma et al.,** **1987 [32]**	**The Netherlands**	51 people with HD	53 (range 26–78)	Cohort study
**Simonin et al.,** **2013 [33]**	**France**	80 people with HD 41 males 39 females	Not reported (NR)	Cohort study
**Marder et al., 2009 [34]**	**United States of America (USA)**	People with HD recruited from Prospective Huntington at Risk Observational study (PHAROS) 1001 participants from PHAROS study 675 HD individuals initially completed the National Cancer Institute FFQ 435 participants with non-expanded CAG < 37 and 217 participants with expanded CAG ≥ 37 completed the FFQ BMI 23 individuals excluded due to (missing CAG data and clinically definite HD prior to or at time of FFQ)	Non-expanded CAG < 37 44.9 (7.9) Expanded CAG ≥ 37 43.4 (7.7)	Case-Control study
**Mochel et al., 2007 [35]**	**France**	32 HD patients CAG expansion > 36 15 pre-symptomatic 17 symptomatic 21 controls	42 ± 11 (range 28 to 80 years) 37 ± 9.5 (range 27 to 62 years)	Case-Control study

HD: Huntington’s disease, NIH: National Institutes of Health, FFQ: food frequency questionnaire, CAG: cytosine-adenine-guanine, EPA: eicosapentaenoic acid, NR: Not reported.

**Table 2:** Characteristics of studies investigating the association of Mediterranean Diet, caloric intake and dietary pattern on Huntington’s disease.

First Author, Year, and Country	Subjects and Ethnicity, N	Mean Age at Blood Collection ± SD Age Range (Years)	Sample Type	Exposure (Dietary Consumption, Dietary Patterns)	Clinical Outcome, Analysis and Effect Estimation	*p*-Value	Cofounders	Clinical Conclusions
**Marder et al.,** **2013 (USA) [16]**	HD carriers recruited from Prospective Huntington at Risk Observational study (PHAROS) Inclusion of 211 HD carriers with expanded CAG ≥ 37 – asymptomatic at baseline Ethnicity: N/A	25–57 years	Blood sample for DNA analysis	MeDi score based on FFQ food group intake and Caloric Intake	Multiple regression models comparing indicated variables between MeDi tertiles: Median (25th percentile, 75th percentile) **UHDRS** MeDi 0–3: 4 (1, 10) MedDi 4–5: 3 (0,8) MeDi 6–9: 2 (1,6) **Chorea** MeDi 0–3: 0 (0, 3) MedDi 4–5: 0 (0,2) MeDi 6–9: 0 (0,2) Adjusted Hazard ratios from Cox proportional hazard models to predict phenoconversion **Caloric intake** Medium vs. Low: HR = 0.61 95%: 0.19, 1.99 High vs. Low: HR = 1.70 95%: 0.65, 4.43 **MeDi Diet** 4-5 vs.0-3: HR = 1.03 95% CI: 0.41, 2.57 6-9 vs. 0-3: HR = 0.74 95% CI: 0.23, 2.42 Cox proportional hazards models: Association between Individual MeDi components and phenoconversion (Lower than sex-specific median intake of detrimental components (i.e., dairy and meat) and higher than sex-specific median intake of beneficial components (i.e., cereal, fish, fruit, legumes, vegetables, MUFA/SFA, moderate alcohol) were treated as reference group: **Cereal (Low intake)** HR = 1.12 95% CI: 0.50, 2.48 **Dairy (High intake)** HR = 2.36 95% CI: 1.00, 5.57 **Fish (Low intake)** HR = 0.71 95% Cl: 0.29, 1.75 **Fruit (Low intake)** HR = 0.74 95% Cl: 0.30, 1.82 **Legumes (Low intake)** HR = 1.87 95% Cl: 0.75, 4.62 **Meat (High intake)** HR = 0.86 95% Cl: 0.37, 1.98 **Vegetables (Low intake)** HR = 2.05 95% Cl: 0.74, 5.72 **MUFA/SFA (Low intake)** HR = 1.40 95% Cl: 0.61, 3.19 **Alcohol (Moderate intake)** HR = 0.8 195% Cl: 0.36 1.83	0.100 0.590 0.070 0.730 0.790 0.0507 0.460 0.510 0.180 0.720 0.170 0.430 0.610	Age CAG repeat length	Adherence to MeDi affects phenoconversion and effects of BMI and caloric intake in time of phenoconversion
**Rivadeneyra et al., 2016** **(Spain)** **[18]**	98 Spanish HD patients and pre-manifesting HD carriers of the European Huntington Disease Networks Ethnicity: N/A	48 (range 38–60)	NR	Adherence to Mediterranean Diet and nutrition assessed via 3 days dietary record	Multiple logistic regression model: Moderate MeDi adherence vs. Low (reference) as the dependent variable Comorbidity OR = 0.18 95% (0.05, 0.75) UHDRS Motor score OR = 0.90 95% (0.81, 0.99) High MeDi adherence vs. Low (reference) as the dependent variable UHDRS Motor score OR = 0.81 95% (0.66, 0.98)	0.018 * 0.041 * 0.033 *	Gender Age Comorbidity UHDRs motor PBAs Dysphagia Physical activity BMI WHtR	Moderate MeDi adherence is associated with better quality life, lower motor impairment and low risk of abdominal obesity compared to HD patients with low MeDi adherence
**Puri et al., 2005 (UK) [19]**	RCT Double blind 135 Symptomatic HD patients 67 in Ethyl EPA 68 in placebo group Ethnicity: Caucasians, African/African Americans and Asians	50 ± 9.3 49 ± 9.0	Peripheral blood	2 g/day ethyl EPA or 2 × 500 mg of capsules of liquid paraffin for a year	ANCOVA test: TMS-4 score ethyl-EPA vs. placebo group 6 months 12 months TMS of UHDRS benefit for ethyl-EPA vs. placebo χ^2^ test: TMS-4 improvement at 12 months ethyl-EPA group vs. placebo χ^2^ test: TMS-4 improvement vs. non-improvement for TMS at 12 months for ethyl-EPA group vs. placebo χ^2^ test: Improvement of total motor score	0.061 0.110 0.046 * 0.075 0.048 * 0.097	Age Sex Baseline severity	Ethyl-EPA in the improvement or non-improvement of the TMS-4 score Ethyl-EPA showed stable or improved motor function Relationship between Ethyl-EPA and CAG repeats
**Puri et al.,** **2008 (UK) [21]**	RCT Double blind 34 Symptomatic HD patients 16 in Ethyl-EPA group 18 in placebo group Ethnicity: N/A	51.3 ± 2.5 48.7 ± 2.2	MRI brain scans	2 g/day of Ethyl-EPA or 2 × 500 mg of light liquid paraffin twice daily for a year	Two samples *t* test within the framework of a GLM: Global atrophy 0–12 months Ethyl-EPA (Mean change −0.75257) vs. placebo (Mean change −1.22381) 0–6 months Ethyl-EPA (Mean change -0.3175) vs placebo (Mean change -0.61511) 7–12 months Ethyl-EPA (Mean change -0.53964) vs. placebo (Mean change −0.59875) Voxel Wise analyses: Global atrophy during the first 6 months in Ethyl-EPA vs. placebo group Global atrophy during second 6 months in Ethyl-EPA vs. placebo group	0.067 (NS) 0.050 NS 0.0001 * 0.0001 *	Age Sex	Treatment of Ethyl-EPA showed reduced rate of atrophy over the first 6 months
**Mochel et al.,** **2010 (France) [22]**	6 HD patients with abnormal CAG repeat expansions 5 females 1 male Ethnicity: N/A	NR	Blood and urine samples	Ingestion of triheptanoin 1 g/kg per day divided in four meals	Paired t tests: Compare values of plasma acylcarnitines, serum IGF1 and UHDRs before and after triheptanoin CAG repeats with plasma glutamine Plasma C3-carnitine Pre-treatment vs. post-treatment Serum IGF1 pre-treatment vs. post-treatment	<0.001 * 0.011 * 0.010 *	Sex Age	Triheptanoin treatment produced a non-significant increase in mean UHDRS
**Goetz et al.,** **1990** **(USA) [23]**	10 HD patients in a double blind RCT, crossover design 4 men and 6 women with HD Ethnicity: N/A	51.6 (16.7)	NR	Administration of L-Acetyl carnitine (LACC) dose of 45 mg/kg/day for 1 week	Friedman’s two-way analysis of variance: Comparison of Reaction time between baseline, placebo and LACC treatment groups Comparison of Mini-Mental examination score between baseline, placebo and LACC treatment groups Comparison of Verbal fluency between baseline, placebo and LACC treatment groups	0.0247 * NS NS	NR	Safety and toxicity of LACC
**Cuturic et al.,** **2012 (USA) [24]**	23 institutionalized HD patients 6 HD patients with treated hypocarnitinemia 17 HD patients without hypocarnitinemia Ethnicity: N/A	43.4 (10.3) 51. 9 (7.9)	Blood sample	Comparison of mean follow up values for motor, cognitive and behavioural parameters	Comparison of patients with and without hypocarnitinenmia **UHDRS initial** 52.8 (14.7) vs. 54.5 (17.1) **UHDRS-6 months** 48.0 (14.7) vs.57.2 (17.1) **UHDRS-12 months** 51.2 (14.1) vs. 60.5 (17.2) **UHDRS change 0–6 months** −4.8 (4.8) vs. +2.7 (2.7) **UHDRS change 6–12 months** +3.2 (1.7) vs. 3.2 (1.8) **MMSE initial** 18.3 (2.7) vs. 17.6 (4.8) **MMSE-6 months** 18.3 (3.23) vs. 16.6 (5.1) **MMSE-12 months** 17.5 (2.9) vs. 15.6 (5.1) **MMSE change 0–6 months** 0.0 (0.6) vs. −1.0 (1.0) **MMSE change 6–12 months** −0.8 (0.4) vs. −0.9 (1.0)	0.831 0.254 0.248 <0.0001 * 0.936 0.727 0.446 0.416 0.018 * 0.796	NR	HD patients with hypocarnitinemia may benefit from a low dose of levocarnitine supplementation Improvement of UHDRS and MMSE scores within the first 6 months of carnitine supplementation suggesting a slower rate of progression and MMSE decline
**Tabrizi et al.,** **2003(UK) [25]**	10 HD patients and 3 HD mutation carriers 4 age matched controls Ethnicity: N/A	NR	Blood sample	10 g per day of creatine for 12 months	Mann-Whitney U two tailed test: Mean total motor score (TMS) Functional capacity score Neuropsychology testing score	NS NS NS	NR	Tolerability, safety and efficacy of creatine supplementation
**Auigner et al.,** **2010(USA)** **[26]**	RCT double-blind parallel group, clinical trail 347 early HD patients 51% males 49% females Ethnicity: N/A	18–75 (47.9 years)	Blood sample	Given either coenzyme Q10 (600 mg/day), Remacemide (600 mg/day) or both treatments or a placebo for 30 months and serum Uric Acid (UA) will be assessed from blood sample	Adjusted mean change in assessments over 30 months by baseline UA quintile: Primary outcome TFC: 1st vs. 2nd vs.3rd vs.4th −3.17 (0.34) vs. −2.99 (0.34) vs. −2.95 (0.29) vs. −2.28 (0.29) vs. −2.21 (0.31) Linear trend for UA Total Motor Score 1st vs. 2nd vs.3rd vs.4th 14.27 (1.57) vs. 13.02 (1.52) vs. 11.56 (1.34) vs.12.56 (1.36) vs. 9.70 (1.43) Linear trend for UA	0.030 * 0.034 * 0.070 0.030 *	Gender Study site Baseline age CAG repeat length Baseline value	Association between higher UA levels and slowing of HD progression by measuring TFC Less worsening in total motor scores with increasing UA levels
**Trejo et al.,** **2005 (Mexico)** **[27]**	30 HD patients 70% male 30% female Ethnicity: N/A	46 (range 20-69)	Blood sample	Nutritional supplement 2 cans of 236 mL in addition to diet for 90 days	Wilcoxon’s signed ranks test or paired t-test: Before and after nutritional intervention **UHDRS Scores-Day 0 vs. Day 90** **Total motor score** 64 ± 14 vs. 65 ± 25 **Maximal chorea** 13 ± 6 vs. 14 ± 5 **Behavioural score** 15 ± 13 vs. 10 ± 13 **Independence** 56 ± 28 vs. 57 ± 27 **Functional checklist score** 11 ± 8 vs. 11 ± 8 **Biochemical Indictors-Day 0 vs. Day 90** **Glucose** 90 ± 14 vs.89 ± 12 **Cholesterol** 179 ± 40 vs. 176 ± 38 **Triacylglycerol** 145 ± 92 vs. 136 ± 72 **Albumin** 4 ± 0.4 vs. 4 ± 0.4 **Hemoglobin** 15 ± 2 vs. 15 ± 3 **Haematocrit** 45 ± 8 vs. 44 ± 11 **Total lymphocyte count** 1938 ± 536 vs. 2149 ± 1141	0.720 0.200 0.100 0.510 0.33 NS NS NS NS NS NS NS	NR	Nutritional innervation slightly improved the anthropometric variables in HD patients Nutritional supplementation increased mean energy and nutritional intake by 20% Good tolerability of patients to the nutritional supplements
**Cubo et al.,** **2015 (Spain)** **[28]**	224 Spanish HD patients and carriers from the European HD registry (EHDN) Control group from Spanish population obtained from EHIDE Ethnicity: N/A	47.41 ± 14.26	NR	Dietary intake and nutritional intake via 3-day record and 24 h FFQ	BMI in pre-manifest vs. manifest HD patients 23.00 (26.25–21.13) vs.23.83 (27.25–21.36) Caloric intake in pre-manifest vs. manifest HD patients 1893± 599.58 vs.2084.25 ± 701.71 Correlation between caloric intake and motor and cognitive UHDRS Association between Mediterranean diet adherence and motor and cognitive UHDRS Dietary factors comparison of severe vs. mild-moderate HD Higher Caloric intake: 1950.62 ± 615.93 vs.2178.53 ± 762.22 Higher fat intake Higher vitamin C Higher vitamin A Higher vitamin E Higher thiamine Higher riboflavin Higher pantothenate Higher pyridoxine Higher biotin Higher calcium Higher phosphorus Higher potassium Higher magnesium Higher iron Higher copper Binary logistic regression with advanced vs. mild moderate HD as dependent variable: Intake of water-soluble vitamins OR = 2.08 95% CI: 1.12–3.85 Minerals OR = 1.86 95% CI: 1.12–3.19	0.330 0.120 NS NS 0.020 * 0.020 * 0.020 * 0.020 * 0.030 * 0.0002 * 0.010 * 0.003 * 0.002 * 0.005 * 0.020 * 0.0028 * 0.010 * 0.002 * 0.002 * 0.001 * 0.005 * 0.020 * 0.020 *	NR NR NR NR NR NR NR Age Gender Education Physical exercise Intake of supplements	Analyse association of nutritional factors with HD severity Adequate dietary intake prevents weight loss in patients with advanced HD but it is not associated with better functional state
**Trejo et al.,** **2004(Mexico) [29]**	25 HD patients 25 age and sex matched controls Ethnicity: N/A	46 ± 12 (21–70) 46 ± 8 (23–72)	Blood sample	Carbohydrate, protein and fat intake via a 3- day FFQ and anthropometric and biochemical indicators	Independent samples *t* test: HD patient’s vs. Controls **HD patient’s vs. Controls** **Mean** **±** **SD** **Weight** 58.2 ± 10.01 vs.66.7 ± 10.1 **BMI** 22.2 ± 2.4 vs.24.6 ± 1.5 **Body fat** 27.6 ± 8.9 vs.37.9 ± 7.1 **Biochemical indictors** **HD patient’s vs. controls** **Mean** **±** **SD** **Albumin** 3.98 ± 0.37 vs.4.04 ± 0.41 **Hemoglobin** 15.10 ± 1.63 vs.15.49 ± 2.44 **Hematocrit** 45.36 ± 4.07 vs.47.79 ± 7.85 **Total lymphocyte count** 1907.68 ± 523.27 vs. 1971.75 ± 456.10 **Glucose** 90.00 ± 11.90 vs. 91.05 ± 10.60 **Cholesterol** 194 ± 44.70 vs. 215.35 ± 42.98 **Triacylglycerol** 193.20 ± 101.94 vs. 208.45 ± 92.19 **Diet indictors** **HD patient’s vs. Controls** **Mean** **±** **SD** **Energy intake** 2325.1 ± 551.4 vs. 1948.4 ± 270.2 **Carbohydrate** **Intake** 330.1 ± 98.4 vs. 252.1 ± 72.6 **Protein intake** 92.3 ± 36.0 vs.80.2 ± 18.1 **Fat intake** 71.7 ± 20.2 vs. 68.3 ± 18.0 Chi-square test: Association of variables in HD patient’s **Weight-loss** 32% **Increased appetite** 36% **Decreased appetite** 4%	0.0047 * 0.0001 * 0.00004 * 0.637 0.526 0.186 0.668 0.759 0.116 0.605 0.003 * 0.003 * 0.14 0.5 0.026 * 0.046 * NS	NR	Nutritional status, anthropometric, biochemical indictors, energy and macronutrient intake to determine indicators altered in HD patients and nutritional requirements to improve their quality of life
**Chen et al.,** **2015(Taiwan) [30]**	30 HD patients 19 men and 11 women 23 controls 12 men and 11 women 14 HD out of 30 HD patients (2 yr. pilot study) 22 HD patients (1 yr. follow up) Ethnicity: Asian	44.7 ± 11.4 42.26 ± 2.6	Arterial blood sample Venous blood sample	Citrulline blood levels, as a marker of urea cycle deficiency in a high protein diet	Pearson’s correlation: Between citrulline concentration in HD patients over 2 years follow up with: **UHDRS Motor Score** R = −0.1584 **Functional capacity** R = 0.06484 **Independence score** R = 0.1193	0.0985 0.5010 0.2144	NR	Blood citrulline concentration – a marker of urea cycle deficiency following a HPD was not associated with HD progression
**Morales et al.,** **1989** **(Venezuela)** **[31]**	18 choreic patients 31 offspring (1st generation) 19 individuals (2nd generation) 7 individuals (3rd generation) 40 controls Ethnicity: N/A	15 to 57 years 12 to 72 years 14 to 45 years 18 to 32 years 14 to 72 years	Fasting blood samples	Nutritional status (daily meal frequency, meal schedule, food quantity and snack intake, energy consumption vegetables, animal proteins, total fat cholesterol and carbohydrates, iron, vitamins A, C, niacin levels and essential amino acids)	ANOVA: comparing amino acid intake in control, choreic patients, 1st generation, 2nd generation and 3rd generation Nutrients and energy provided by food intake in control, choreic patients, 1st generation, 2nd generation and 3rd generation **Control** Calories 1420.62 ± 101.69 Animal protein 38.94 ± 3.42 Vegetal protein 14.62 ± 1.26 Lipids 56.80 ± 5.50 Carbohydrates 186.25 ± 13.37 **Choreic patients** Calories 1731.33 ± 191.32 Animal protein 35.61 ± 15.15 Vegetal protein 19.88 ± 2.38 Lipids 60.05 ± 7.61 Carbohydrates 259.33 ± 32.19 **1st generation** Calories 1470.26 ± 102.16 Animal protein 43.12 ± 4.89 Vegetal protein 14.81 ± 1.42 Lipids 63.25 ± 27.68 Carbohydrates 225.46 ± 27.68 **2nd generation Calories** 1481.57 ± 128.72 Animal protein 40.52 ± 4.47 Vegetal protein 12.05 ± 1.17 Lipids 59.52 ± 5.56 Carbohydrates 196.10 ± 18.65 AVONA comparing Vitamin C between groups–choreic patients were deficient AVONA comparing Niacin between groups–choreic patients were deficient	NS NS NS NS NS <0.01 * <0.01 *	Age Gender Gender Age Age	
**Buruma et al.,** **1987 (The Netherlands) [32]**	51 HD patients Ethnicity: N/A	53 (range 26-78)	NR	Eating, drinking and smoking habits over a period of 10 years before the age of onset	Spearman’s rank correlation test between overall age at onset (AOAS) and consumption of: **Alcohol** r = 0.09 **Milk intake** r = -0.30 **Coffee** r = 0.07 **Fruit juice** r = -0.10 **Tea** r = -0.09 **Cheese** r = 0.14 **Fish** r = 0.01 Spearman’s rank correlation test between Age at onset of psychological changes (AOPC) and consumption of: **Alcohol** r = 0.11 **Milk intake** r = −0.29 **Coffee** r = 0.00 **Fruit juice** r = −0.16 **Tea** r = −0.25 **Cheese** r = 0.12 **Fish** r = −0.04 Spearman’s rank correlation test between Age at onset of involuntary movements (AOIM) and consumption of: **Alcohol** r = 0.10 **Milk intake** r = −0.29 **Coffee** r = −0.04 **Fruit juice** r = −0.04 **Tea** r = −0.05 **Cheese** r = 0.20 **Fish** r = 0.10 Spearman’s rank correlation test between Rate of progression (RP) and consumption of: **Alcohol** r = −0.09 **Milk intake** r = 0.19 **Coffee** r = 0.00 **Fruit juice** r = −0.02 **Tea** r = 0.31 **Cheese** r = 0.17 **Fish** r = 0.12	0.520 0.030 0.610 0.500 0.520 0.320 0.940 0.470 0.050 0.980 0.280 0.090 0.420 0.800 0.490 0.040 0.750 0.790 0.720 0.170 0.470 0.520 0.190 0.990 0.920 0.030 0.240 0.420		
**Simonin et al., 2013 (France) [33]**	80 HD patients 41 males 39 females Ethnicity: N/A	NR	NR	Mean daily caffeine intake (coffee, tea, chocolate and soda) assessed retrospectively before and after disease onset using dietary survey	Student t test: Comparison of two groups Caffeine intake < 190 mg/d before disease onset vs. Caffeine intake ≥ 190 mg/d before disease onset **AAO:** 49.5 (12.7) vs. 45.4 (10.0) **Annual IS decline** −4.9 (7.1) vs. −2.1 (6.9) **Annual TFC decline** −1.1 (1.4) vs.−0.8 (1.4) **Annual motor UHDRS decline** +7.1 (8.9) vs. +3.7 (7.6) **Annual chorea impairment** +1.6 (4.5) vs. +1.1 (3.6) Multivariable linear regression: **Higher caffeine consumption before disease onset (≥190 mg/d vs. <190 mg/d)** **AAO** Regression coefficient: −3.6	0.1144 0.1072 0.3778 0.1028 0.6426 0.0270 *	Gender Smoking status CAG repeat length Disease duration	Higher caffeine consumption before onset was associated with a younger age of onset
**Marder et al.,** **2009 (USA) [34]**	HD carriers (pre-symptomatic) and controls recruited from Prospective Huntington at Risk Observational study (PHAROS) 435 participants with non-expanded CAG <37 217 participants with expanded CAG ≥ 37 Ethnicity: N/A	44.9 (7.9) 43.4 (7.7)	Blood sample	Caloric intake, body mass index (BMI), dietary consumption of macronutrients (carbohydrates, protein, fat intake) assessed via a FFQ	t tests and x^2^ tests: Demographics and measures from FFQ compared in CAG < 37 vs. CAG ≥ 37 groups Carbohydrates 222.7 ± 113.7 vs. 247.5 ± 119.7 Protein 75.7 ± 40.9 vs. 79.0 ± 38.2 Fat 71.3 ± 40.8 vs. 73.3 ± 35.3 Caloric intake CAG < 37 1858 (940) (224-7138) (113.7) and CAG ≥ 37 1994 (901) (423–6654) BMI CAG < 37 28.4 (6.6) (17.9–56.1) and CAG ≥ 37 27.0 (5.4) (16.7–52.0) Mantel-Haenszel tests for trend after dividing variables into 4 quartile groups. Comparing CAG < 37 vs. CAG ≥ 37 groups **Caloric intake** Q1 CAG < 37 29.2 and CAG ≥ 37 16.6 Q2 CAG < 37 23.2 and CAG ≥ 37 28.6 Q3 CAG < 37 23.7 and CAG ≥ 37 27.7 Q4 CAG < 37 23.9 and CAG ≥ 37 27.2 **Carbohydrates** Q1 CAG < 37 29.0 and CAG ≥ 37 17.1 Q2 CAG < 37 23.7 and CAG ≥ 37 28.6 Q3 CAG < 37 23.9 and CAG ≥ 37 28.1 Q4 CAG < 37 23.5 and CAG ≥ 37 26.3 **Proteins** Q1 CAG < 37 26.0 and CAG ≥ 37 21.7 Q2 CAG < 37 25.3 and CAG ≥ 37 25.4 Q3 CAG < 37 25.3 and CAG ≥ 37 26.7 Q4 CAG < 37 23.5 and CAG ≥ 37 26.3 **Fat** Q1 CAG < 37 26.9 and CAG ≥ 37 20.3 Q2 CAG < 37 25.1 and CAG ≥ 37 25.8 Q3 CAG < 37 24.6 and CAG ≥ 37 27.7 Q4 CAG < 37 23.5 and CAG ≥ 37 26.3 Regression between Caloric Intake and CAG repeat length Regression coefficient (SE): 0.26 (0.12) Regression between Caloric Intake and the 5 years probability of HD onset in subjects with CAG ≥ 37 Regression coefficient (SE): 0.024 (0.010)	**0.010** NS NS 0.073 0.006 * 0.013 * 0.019 * 0.220 0.097 0.032 * 0.013 *	Age Gender Education Total caloric intake Age Gender Education Total caloric intake Age Gender Education Total caloric intake Age Gender Education Total caloric intake CAG repeat length Caloric intake	
**Mochel et al.,** **2007 (France) [35]**	32 HD patients CAG expansion > 36 15 pre-symptomatic 17 symptomatic 21 controls Ethnicity: N/A	42 ± 11 (range 28 to 80 years) 37 ± 9.5 (range 27 to 62 years)	Blood and urine samples	Semi-quantitative questionnaire of regular food and beverage consumption to observe caloric intake in HD patients	ANOVA Comparison of means Weight loss determined retrospectively over a 5 years period HD patients (−3.3 ± 4.5 kgs) vs. Controls (−2.8 ± 3.9 kgs) BMI HD patients (22.6 ± 3.0 kg/cm^2^) vs. Controls (24.0 ± 5.0 kg/cm^2^) Daily caloric intake determined from a 3 day and 24 h questionnaire performed at 1-month interval HD patients (2020 ± 530/24 h) vs. Controls (1665 ± 305/24 h) Proteins determined from a 3 day and 24 h questionnaire performed at 1-month interval HD patients (81.3 ± 23.7 gr/24 h) vs. Controls (70.0 ± 14.1 gr/24 h) Lipids HD patients (86.5 ± 18.3 gr/24 h) vs. Controls (65.7 ± 18.0 gr/24) Sugar HD patients (216.0 ± 77.1 gr/24 h) vs. Controls (191.3 ± 48.6 gr/c24 h)	0.001 * 0.217 0.008 * 0.054 0.001 * 0.201	Age BMI	Weight loss starts early in disease Low levels of BCAA correlated with weight loss, disease progression and abnormal repeat expansion size

NS: Non-significant, TFC: Total Functional Capacity, UA: Uric acid, CAG: Cytosine-Adenosine-Guanine, AOIM: Age of onset of involuntary movement, AOPC: Age of onset of psychological changes, RCT: Randomized Controlled Trial, MMSE: Mini-Mental State Examination, UHDRS: Unified Huntington’s Disease Rating Scale, EHDN: European Huntington’s Disease Network, RP: Rate of progression, NR: Not reported, N/A: Not available, BCAA: branched-chain amino acid. Significant *p*-values are indicated in bold *.

## Supplementary Materials

The following are available online at https://www.mdpi.com/2072-6643/12/10/2946/s1, Table S1: PRISMA Checklist, Table S2: Quality assessment of cohort studies using Newcastle-Ottawa, Table S3: Quality assessment of case control studies using Newcastle-Ottawa, Table S4: Quality assessment of randomized trials using Cochrane, Figure S1: Newcastle-Ottawa quality assessment scale case control studies, Figure S2: Newcastle-Ottawa quality assessment scale cohort studies, Figure S3 Cochrane risk of bias tool for randomized controlled trials. Supplementary information S1: Study descriptions.

## Abbreviations

AAO	Age of Onset AD Alzheimer’s Disease
AIMS	Abnormal involuntary movement
ALS	Amyotrophic Lateral Sclerosis
AOIM	Age of Onset of Involuntary Movements
AOPC	Age of Onset of Psychological Changes
BMI	Body Mass Index
BBB	Blood Brain Barrier
CAG	Cytosine Adenosine Guanine
CNS	Central Nervous System
DA	Dopamine
EHDN	European Huntington’s Disease Network
FFQ	Food Frequency Questionnaire
GABA	Gamma-Aminobutyric acid
HD	Huntington’s Disease
HPD	High Protein Diet
HTT	Huntingtin
IS	Independence Score
ITT	Intent to treat
LACC	L-acetyl carnitine
MD	Mediterranean Diet
mHTT	Mutant Huntingtin
MMSE	Mini Mental State Examination
MUFA	Monounsaturated Fatty Acids
NR	Not Reported
3-NP	3-Nitropmiddleionic acid
NS	Non-significant
PD	Parkinson’s Disease
PP	Protocol Violation
PUFA	Polyunsaturated Fatty Acids
RCT	Randomized Controlled Trial
RP	Rate of progression
SFA	Saturated Fatty Acids
TFC	Total Functional Capacity
TMS	Total Motor Score
UA	Uric Acid
UHDRS	Unified Huntington’s Disease Rating Scale
