# Supplementary material for: Dietary Intake, Mediterranean Diet Adherence and Caloric Intake in Huntington’s Disease: A Review
Source: Nutrients. 2020 Sep 25;12(10):2946. doi: 10.3390/nu12102946 (PMC7601299; doi:10.3390/nu12102946)
Supplement: Supplementary file 1 [file nutrients-12-02946-s001.zip › S1_Table_PRISMA checklist_corrected.doc]

Table S1 PRISMA checklist

| **Section/topic** | **#** | **Checklist item** | **Reported on page #** |
| --- | --- | --- | --- |
| **TITLE** | | |  |
| Title | 1 | Identify the report as a systematic review, meta-analysis, or both. | 1 |
| **ABSTRACT** | | |  |
| Structured summary | 2 | Provide a structured summary including, as applicable: background; objectives; data sources; study eligibility criteria, participants, and interventions; study appraisal and synthesis methods; results; limitations; conclusions and implications of key findings; systematic review registration number. | 1 |
| **INTRODUCTION** | | |  |
| Rationale | 3 | Describe the rationale for the review in the context of what is already known. | 2-3 |
| Objectives | 4 | Provide an explicit statement of questions being addressed with reference to participants, interventions, comparisons, outcomes, and study design (PICOS). | 3 |
| **METHODS** | | |  |
| Protocol and registration | 5 | Indicate if a review protocol exists, if and where it can be accessed (e.g., Web address), and, if available, provide registration information including registration number. | Not applicable |
| Eligibility criteria | 6 | Specify study characteristics (e.g., PICOS, length of follow-up) and report characteristics (e.g., years considered, language, publication status) used as criteria for eligibility, giving rationale. | 3-4 |
| Information sources | 7 | Describe all information sources (e.g., databases with dates of coverage, contact with study authors to identify additional studies) in the search and date last searched. | 3-4 |
| Search | 8 | Present full electronic search strategy for at least one database, including any limits used, such that it could be repeated. | 3-4 |
| Study selection | 9 | State the process for selecting studies (i.e., screening, eligibility, included in systematic review, and, if applicable, included in the meta-analysis). | 3-4 |
| Data collection process | 10 | Describe method of data extraction from reports (e.g., piloted forms, independently, in duplicate) and any processes for obtaining and confirming data from investigators. | 3-4 |
| Data items | 11 | List and define all variables for which data were sought (e.g., PICOS, funding sources) and any assumptions and simplifications made. | 3-4 |
| Risk of bias in individual studies | 12 | Describe methods used for assessing risk of bias of individual studies (including specification of whether this was done at the study or outcome level), and how this information is to be used in any data synthesis. | 3-4 |
| Summary measures | 13 | State the principal summary measures (e.g., risk ratio, difference in means). | Not applicable |
| Synthesis of results | 14 | Describe the methods of handling data and combining results of studies, if done, including measures of consistency (e.g., I^2^) for each meta-analysis. | Not applicable |

Page 1 of 2

| **Section/topic** | **#** | **Checklist item** | **Reported on page #** |
| --- | --- | --- | --- |
| Risk of bias across studies | 15 | Specify any assessment of risk of bias that may affect the cumulative evidence (e.g., publication bias, selective reporting within studies). | Supplementary data  5-20 |
| Additional analyses | 16 | Describe methods of additional analyses (e.g., sensitivity or subgroup analyses, meta-regression), if done, indicating which were pre-specified. | Not applicable |
| **RESULTS** | | |  |
| Study selection | 17 | Give numbers of studies screened, assessed for eligibility, and included in the review, with reasons for exclusions at each stage, ideally with a flow diagram. | 3-4 |
| Study characteristics | 18 | For each study, present characteristics for which data were extracted (e.g., study size, PICOS, follow-up period) and provide the citations. | 10-13 |
| Risk of bias within studies | 19 | Present data on risk of bias of each study and, if available, any outcome level assessment (see item 12). | Not applicable |
| Results of individual studies | 20 | For all outcomes considered (benefits or harms), present, for each study: (a) simple summary data for each intervention group (b) effect estimates and confidence intervals, ideally with a forest plot. | 10-41 |
| Synthesis of results | 21 | Present results of each meta-analysis done, including confidence intervals and measures of consistency. | Not applicable |
| Risk of bias across studies | 22 | Present results of any assessment of risk of bias across studies (see Item 15). | Supplementary data  5-20 |
| Additional analysis | 23 | Give results of additional analyses, if done (e.g., sensitivity or subgroup analyses, meta-regression [see Item 16]). | Not applicable |
| **DISCUSSION** | | |  |
| Summary of evidence | 24 | Summarize the main findings including the strength of evidence for each main outcome; consider their relevance to key groups (e.g., healthcare providers, users, and policy makers). | 10-41 |
| Limitations | 25 | Discuss limitations at study and outcome level (e.g., risk of bias), and at review-level (e.g., incomplete retrieval of identified research, reporting bias). | 39-40 |
| Conclusions | 26 | Provide a general interpretation of the results in the context of other evidence, and implications for future research. | 39-41 |
| **FUNDING** | | |  |
| Funding | 27 | Describe sources of funding for the systematic review and other support (e.g., supply of data); role of funders for the systematic review. | 41 |

*From:* Moher D, Liberati A, Tetzlaff J, Altman DG, The PRISMA Group (2009). Preferred Reporting Items for Systematic Reviews and Meta-Analyses: The PRISMA Statement. PLoS Med 6(7): e1000097. doi:10.1371/journal.pmed1000097

For more information, visit: **www.prisma-statement.org**.

Figure S1 Newcastle-Ottawa quality assesment scale case control studies

Figure S2 Newcastle-Ottawa quality assesment scale cohort studies

Table S2 Quality assessment of cohort studies using Newcastle-Ottawa

|  | **Selection** | | | | **Comparability** | | **Outcome** | | | **Final score** |
| --- | --- | --- | --- | --- | --- | --- | --- | --- | --- | --- |
| **Author and year** | **Representativeness of the exposure** | **Selection of non-exposed** | **Ascertainment of exposure** | **Outcome not present at start** | **Main factor** | **Additional factor** | **Assessment outcome** | **Follow up length** | **Adequacy of follow up** |  |
| **Trejo et al., 2005** | * | - | * | * | - | - | * | * | - | 5 |
| **Cuturic et al., 2012** | * | - | * | * | - | - | * | * | * | 6 |
| **Simonin et al., 2013** | * | - | * | * |  |  | * | * | * | 6 |
| **Cubo et al. 2015** | * | * | * | * | * | * | * | - | - | 7 |
| **Buruma et al., 1987** | * | - | * | * | - | - | * | * | - | 5 |
| **Marder et al., 2013** | * | * | * | * | * | * | * | * | * | 9 |
| **Rivadeneyra et al., 2016** | * | * | * | * | * | * | * | - | - | 7 |
| **Chen et al., 2015** | * | * | * | * | * | * | * | * | * | 9 |
| **Marder et al., 2009** | * | * | * | * | * | * | * | * | - | 8 |
| **Mochel et al., 2010** | - | - | * | * | * | * | * | * | - | 6 |

***Selection***

1) Representativeness of the exposed cohort: a) truly representative of the average in the community, b) somewhat representative of the average in the community, c) selected group of users e.g. nurses, volunteers and d) no description of the derivation of the cohort
2) Selection of the non-exposed cohort: a) drawn from the same community as the exposed cohort and b) drawn from a different source and c) no description of the derivation of the non-exposed cohort
3) Ascertainment of exposure: a) secure record (e.g. surgical records), b) structured interview, c) written self-report and e) no description
4) Demonstration that outcome of interest was not present at start of study: a) yes and b) no

***Comparability***

1) Comparability of cases and controls on the basis of the design or analysis: a) study controls for _______________ (Select the most important factor) and b) b) study controls for any additional factor.

***Outcome***

1) Assessment of outcome: a) independent blind assessment, b) record linkage, c) self-report , d) no description
2) Was follow-up long enough for outcomes to occur: yes (select an adequate follow up period for outcome of interest) and b) no.
3) Adequacy of follow up of cohorts: a) complete follow up - all subjects accounted for, b) subjects lost to follow up unlikely to introduce bias - small number lost - > ____ % (select an adequate %) follow up, or description provided of those lost, c) follow up rate < ____% (select an adequate %) and no description of those lost and d) no statement

Table S3 Quality assessment of case controls studies using Newcastle-Ottawa

|  | **Selection** | | | | **Comparability** | **Exposure** | | | **Total Quality Score** |
| --- | --- | --- | --- | --- | --- | --- | --- | --- | --- |
| **Author and year** | **Case definition adequate** | **Representativeness of the cases** | **Selection of Controls** | **Definition of Controls** | **Comparability of cases and controls on the basis of the design or analysis** | **Ascertainment of exposure** | **Same method of ascertainment for cases and controls** | **Non-Response rate** |  |
| **Mochel et al., 2007** | * | * | * | * | * | * | * | * | 8 |
| **Morales et al., 1988** | * | * | * | * | * | - | * | * | 7 |
| **Trejo et al., 2004** | * | * | * | * | * | * | * | * | 8 |
| **Tabrizi et al., 2003** | * | * | * | * | * | * | * | * | 8 |

***Selection***

5) Is the case definition adequate: a) yes, with independent validation, b) yes, eg. Record linkage or based on self reports and c) no description
6) Representativeness of the cases: a) consecutive or obviously representative series of cases and b) potential for selection biases or not stated.
7) Selection of Controls: a) community controls, b) hospital controls and c) no description.
8) Definition of Controls: a) no history of disease and b) no description of source.

***Comparability***

2) Comparability of cases and controls on the basis of the design or analysis: a) study controls for _______________ (Select the most important factor) and b) b) study controls for any additional factor.

***Exposure***

4) Ascertainment of exposure: a) secure record (eg surgical records), b) structured interview where blind to case/control status, c) interview not blinded to case/control status, d) written self-report or medical record only and e) no description.
5) Same method of ascertainment for cases and controls: a) yes and b) no.
6) Non-Response rate: a) same rate for both groups, b) non respondents described and c) rate different and no designation

Table S3 Cochrane risk of bias tool for randomized controlled trials

Table S4 Quality assessment of randomized trials using Cochrane

|  | **Selection bias** | | | | **Performance bias** | **Detection bias** | **Attrition bias** |
| --- | --- | --- | --- | --- | --- | --- | --- |
| **Author and year** | **Random sequence generation** | **Allocation concealment** | **Selective reporting** | **Other sources of bias** | **Blinding (participants and personal)** | **Blinding (outcome assessment)** | **Incomplete outcome data** |
| **Puri et al., 2005** |  |  |  |  |  |  |  |
| **Puri et al., 2008** |  |  |  |  |  |  |  |
| **Goetz et al., 1990** |  |  |  |  |  |  |  |
| **Auinger et al., 2010** |  |  |  |  |  |  |  |

**High risk of bias**

**Low risk of bias**

**Unclear risk of bias**

**Supplementary information S1**

**Study descriptions.**

Eapentaenoic acid is a derivative of the omega-3 fatty acid eicosapentaenoic acid (EPA). Even though formulated as a fish oil drug, Ethyl-EPA is similar to fish oil dietary supplements but with better-controlled ingredients [19].

A study by Puri *et al*., 2005*.* investigated the effect of Ethyl-EPA in people with HD in terms of their improvement or stability in their motor function, using the total motor function-4 (TMS-4) a sub-scale of the UHDRS. The study recruited 135 people with HD however, only a total of 121 patients completed the 12-month study period. The study participants were randomized into a double blind placebo controlled trial, and 2g/day of Ethyl-EPA vs placebo were given to study participants. The results showed that in the intent-to-treat cohort (ITT), there was no significant difference between ethyl-EPA and placebo and TMS-4. In the per protocol cohort (PP), however, the ethyl-EPA group performed better than the placebo group on TMS-4 at 12 months (p<0.05). A beneficial effect for ethyl-EPA vs placebo (p=0.046) was also identified for the TMS of the UHDRS. Furthermore, after 12 months a non-significant improvement in the TMS-4 score (p=0.075) was observed between the ethyl-EPA vs placebo groups. The TMS and TMS-4 subscale showed the highest sensitivity to ethyl-EPA as observed in people with HD.

An additional study by Puri *et al*., 2008*.* investigated the effect of ethyl-EPA in reducing cerebral atrophy in patients with HD. Thirty-four symptomatic people with HD were recruited into the randomized, double blind study. Patients, were then separated into two groups: 16 participants in the Ethyl-EPA supplementation group and 18 in the placebo group. During the time period of 0-12 months, the change in cerebral atrophy between the ethyl-EPA and placebo group was non-significant (p=0.067), and it was also non-significant for the period of 7-12 months. However, the 16 participants in the ethyl-EPA group showed significant reductions in brain atrophy in the caudate nucleus and the posterior thalamus during the first 6 months of treatment compared to the placebo group (p<0.05). Furthermore, the Voxel-Wise analysis revealed a significant reduction in global atrophy in the ethyl-EPA group compared to the placebo group, during the first 6 months of treatment (p<0.0001) and the differences were mainly observed in the lateral ventricles.

A study by Mochel *et al*., 2010 studied the tolerability of triheptanoin supplements in a short term clinical trial. Triheptanoin is a triglyceride that is composed of three seven-carbon fatty acids. Six people with HD were recruited for the study, and participants were given 1g/kg her day of triheptanoin, for 4 days. All people with HD tolerated triheptanoin well. Pre-treatment UHDRS scores and post-treatment UHDRS scores were compared but they did not show any significant difference. The study also investigated CAG repeat length and metabolic measurement at baseline, but there was no association between the two variables, except for the inverse correlation with plasma glutamine (p<0.001). The study also found a significant increase in serum Insulin like growth factor-1 (IGF-1) in all people with HD (p=0.010) following treatment with triheptanoin, and demonstrated a significant improvement of energy metabolism in these patients.

A study by Trejo *et al*., 2005 investigated the effects of oral nutritional supplements in 30 people with HD. The nutritional supplements used in the study provided to patients an extra 473 kcal to their diet, and were taken for a period of 90 days. The biochemical indicators did not show any significant changes during the study and were within the normal range. Moreover, the UHDRS was used to assess and score, total motor scores, maximal chorea, behavioural score, independence and functional checklist score of people with HD. However, there was no significant changes observed in people with HD from day 0 vs day 90. Therefore, in this study, diet and biochemical indicators did not have an effect on the UHDRS scores. Nevertheless, the use of nutritional supplements showed a small improvement and stabilization of the anthropometric indicators measured. Following the 90-day study period, 22 patients showed significant changes in anthropometric measurements such as increased body weight (p <0.050), BMI (p <0.050), mid-arm (p <0.050) and arm muscle (p <0.050) circumference and body fat percentage (p <0.05).

Carnitine is a regulator of lipid metabolism; it plays a role in transporting long chain fatty acids into the mitochondrial matrix, it facilitates beta-oxidation and energy production through the Krebs cycle. Cuturic *et al*., 2013*.* measured serum carnitine levels in 23 institutionalized people with HD. Six of these patients were treated with carnitine as their initial serum carnitine concentration were below the normal reference range and 17 were not treated with carnitine as their initial serum carnitine concentration was within the normal reference range. A comparison between patients with hypocarnitinemia that were treated with carnitine supplementation and patients without hypocarnitinemia which were not treated with carnitine supplementation showed that there was no significant differences between the UHDRS and Mini Mental State Examination (MMSE) scores. During the first 6 months of carnitine supplementation, an improvement was seen in the UHDRS scores (p<0.0001) of the six people with HD who were treated with carnitine. Furthermore, during the same month period, the rate of MMSE decreased (p<0.0001) in the group receiving carnitine. The final 6 months of follow-up did not show a difference in UHDRS scores, MMSE decline, falls and violent episodes between the two

Acetyl-L-carnitine (LACC) is an ester of the trimethylated amino acid L-carnitine.

The body naturally produces L-carnitine. Carnitine plays a role in energy production from long chain fatty acids in the mitochondria [36]. Goetz *et al*., 1990 studied the effect of L-Acetyl carnitine (LACC) in 10 people with HD for a period of 1 week. Baseline measurements, were obtained through MMSE, abnormal involuntary movement scale (AIMS), verbal fluency and reaction time from people with HD before placebo and LACC was given. The placebo given to people with HD was a combination of neuroleptic medication (seven patients received haloperidol 12.5 mg/day and one patient received fluphenazine 8 mg/day). A 2-week drug free period separated the placebo and LACC treatment periods. No significant differences were observed in the MMSE score and in verbal fluency between baseline, placebo and LACC treatment periods. The only difference observed between the three groups was the reaction time (p=0.0247).

A study by Tabrizi *et al*., 2003 investigated the effect of creatine therapy in HD, looking at both clinical and Magnetic Resonance Spectroscopy (MRS) findings for 1 year. A total of 13 people with HD, 3 unaffected and 10 affected patients were recruited into the study and 4 age matched controls, and all were given 10 g per day of creatine. No significant change was observed in the mean total motor score, functional capacity (TMS) and neuropsychology testing between baseline and the 1-year period for either group with creatine. MRS studies indicated that creatine was elevated in brain and muscle. This was assessed by N-acetylaspartate/creatine and phosphocreatine (PCr)/ATP ratios. In the occipital lobe, the NAA/Cr at 6 months was (p<0.010) and (p<0.050) at 12 months. In skeletal muscle the PCr/ATP and PCr/Pi at 6 months was (p<0.030) and (p<0.030) respectively.

A study by Auinger *et al*., 2010 investigated the relationship between uric acid (UA) levels and progression of HD by looking at the functional decline of HD. The study recruited 347 People with HD from the CARE-HD. At each visit patients were assessed using the UHDRS and were given either coenzyme Q10 (600 mg/day), Remacemid (600 mg) or both treatments or a placebo for a period of 30 months. Blood samples were taken to measure the serum UA. Study participants in the highest UA quintile had a low mean total motor score at baseline compared to participants in the 1st and 3rd quintiles. In addition, an association was found between baseline UA quintile and a change in the Total Functional Capacity (TFC) over a period of 30 months. The decrease in mean TFC scores over the 30-month period decreased from the lowest to the highest quintile (3.17, 2.99, 2.95, 2.28, 2.21 respectively (p=0.03)). Increasing UA levels also lead to less worsening in total motor scores, the adjusted mean worsening in scores being 14.27, 13.03, 11.56, 12.15, 9.70 from the lowest to highest quintile respectively (p=0.070). The other functional domains such as cognitive, behavioural, depression and neuropsychological were not significantly associated with UA quintile. Therefore, based on this study there is a high association between baseline UA concentration and slower progression of HD.

**Dietary intake and HD**

Another study by Mochel *et al*., 2007 examined the possible mechanisms responsible for weight loss in HD and also to aimed to identify plasma markers which can be traced during HD disease progression. Thirty-two people with HD with an expanded CAG repeat, 15 pre-symptomatic and 17 symptomatic were included, and 21 controls. The study found evidence of an early hypermetabolic state in HD. Weight loss was studied for a period of 5-years and was compared between people with HD and controls. Significant weight loss was observed in people with HD (p<0.001). This difference continued to remain significant when men and women were analysed separately (p=0.002) and (p=0.003). BMI was compared between people with HD and controls (p=0.217). In men with HD, the BMI was lower compared to controls and total caloric intake was inversely correlated with weight (p=0.029) and lean body mass (p=0.0047), indicating a hypermetabolic state in HD. Lastly, caloric intake, proteins, lipids and sugar were compared between people with HD and controls using a 3 day and a 24 hr FFQ. Marder *et al*., 2009 investigated the caloric intake, dietary intake and BMI of HD participants in the Prospective Huntington at Risk Observational Study (PHAROS). Participants were grouped into either CAG ≥ 37 and CAG <37 groups. A total of 435 participants with CAG <37 and 217 with CAG ≥ 37 were included and completed the FFQ. A food frequency questionnaire (FFQ), was used to obtain data regarding dietary intake from both groups. Other relevant data such as BMI, physical activity, motor and chorea UHDRS scores was also collected. Macronutrient intake, as calculated from the FFQ however, it did not show any difference in the distribution of macronutrients between the CAG ≥ 37 and CAG <37 groups, with the exception of carbohydrate intake which was higher in the CAG ≥ 37 group (p-value 0.010). The study also investigated the relationship between CAG repeat length, BMI, caloric intake and estimated physical activity. Caloric intake (p-value 0.0320) was significantly correlated with CAG repeat length and estimated 5-year probability for HD onset (p-value 0.013) for the expanded CAG ≥ 37 group. There was no significant correlation between BMI and CAG repeat length. The correlation between caloric intake and increase in the 5 year probability of disease onset is likely due to a higher consumption of calories, for individuals to maintain their weight during the pre-manifesting phase of HD but at some point they are unable to maintain energy balance. In contrast, early and mid-stage people with HD who were provided with adequate caloric intake in a controlled environment were able to maintain energy balance [37].

The association between caffeine consumption and age of onset (AAO) or functional/motor decline in HD was investigated by Simonin *et al*., 2013. There was a strong correlation between average caffeine intake before and after disease onset (p<0.001). There was no significant association between UHDRS motor score and caffeine intake (p=0.21) when adjusting for disease duration, smoking and gender. However, earlier AAO was associated with an increased caffeine consumption (≥190 mg/day) before disease onset (p=0.0041). Furthermore, alcohol did not influence AAO in this study. Lastly, there was no significant change observed between the caffeine intake <190 mg/day group and the caffeine intake ≥190 mg/day group in decline of independence scale (IS), TFC, total motor decline, and chorea impairment.

Cubo *et al*., 2015 studied the impact and relationship between nutritional status and HD severity in a Spanish cohort of patients and carriers from the European Huntington’s Disease Network (EHDN registry). This included 224 Spanish People with HD, carriers, and control group from the Spanish population obtained from the Spanish National Survey known (EHIDE). Food consumption, caloric and nutritional intake was assessed in people with HD after disease onset, using validated questionnaires. Caloric intake (p=0.120) and BMI (p=0.330) were similar when pre-manifesting and manifested people with HD were compared. After stratification of participants based on the intake of nutritional supplements and living status, only the UHDRS cognitive score was significantly associated with increasing caloric intake (p<0.0001) in institutionalized people with HD. The study also investigated the association of dietary intake with HD severity. Severe people with HD had a higher caloric intake (p=0.020) compared to mild-moderate HD patients. BMI (p=0.330) and dietary intake were similar between HD severity groups, except for high intake of fat (p=0.020), vitamin C (p=0.020), vitamin A (p=0.030), vitamin E (p=0.0002), thiamine (p=0.010), riboflavin (p=0.003), pantothente (p=0.002), pyridoxine (p=0.005), biotin (p=0.020), calcium (p=0.002), phosphorus (p=0.010), potassium (p=0.002), magnesium (p=0.002), iron (p=0.001) and copper (p=0.005) were all higher in the severe HD group compared to the mild-moderate group. The regression model, indicated that a high intake of water soluble vitamins (p= 0.020) and minerals (p=0.020) were associated with advanced HD. However, an association between MD adherence, motor and cognitive UHDRS was not significant.

A study by Morales *et al*., 1989 investigated nutritional evaluation that included the analysis of nutritional, caloric intake, clinical and anthropometric evaluation between people with HD vs 1st, 2nd and 3rd generation descendants of people with HD, and controls. A comparison between essential and non-essential amino acid intake was compared between people with HD (choreic patients) vs HD descendant’s vs controls, however no significant difference was found between amino acids and the different groups. Nutritional and caloric intake was investigated between People with HD vs HD descendants vs controls. The only observation was that choreic patients had more calories compared to HD descendants and controls. Positive correlations (p<0.010) were identified between proteins, lipids, carbohydrates and calorie consumption between choreic patients, controls and 1st and 2nd generation descendants. No correlation was found for macronutrient intake and calorie consumption in 3rd generation descendants. Micronutrient intake in the choreic group showed differences, which were significant. Iron was identified to be less than the recommended intake 14mg/day in all groups. A significant difference by gender (p<0.010) in iron intake was observed and indicated that women with HD were more likely to have an iron deficient diet. Additional micronutrients, such as Vitamin C (p<0.010) and Niacin (p<0.010) were deficient in choreic patients.

Buruma *et al*., 1987 investigated which lifestyle factors such as smoking, dietary habits and drinking may influence age of onset and the rate of progression in HD. Fifty-one Dutch HD patients were recruited into the study and their lifestyle habits were examined for a period of 10 years before the age of onset. In terms of the dietary factors that may influence age at onset no significant relationship was identified for alcohol, coffee, fruit juice, tea, cheese and fish p= 0.470, p=0.980, p=0.280, p=0.090, 0.420 and p=0.800 respectively. However, the consumption of milk showed significance (p=0.050), in that previous high milk intake was associated with earlier AOAS.

**MD adherence and HD**

A study by Marder *et al*., 2013 investigated whether adherence to the MD, BMI and caloric intake affected the period of phenoconversion in people with HD. A total of 211 patients with an expanded ≥37 CAG repeat participated in the study. Fifteen participants were excluded from the study based on if they had phenoconverted when the FFQ was complete or for those individuals who had there last visit. Higher BMI was associated with lower adherence to MD (p=0.020). Phenoconverters are typically older and have slightly higher CAG repeat length compared to participants who did not phenoconvert during the study period. The study found that both age and CAG repeat length were associated with phenoconversion but there was no association between MD adherence and phenoconversion (p= 0.140). There was a trend that higher caloric intake but not BMI was a risk factor for phenoconversion (p=0.0351). In order to identify if MD plays a role in phenoconversion, each of its dietary components was analysed independently. Only the increased consumption of dairy products was associated with a greater risk of early phenoconversion, but not significantly (p=0.051).

Trejo *et al*., 2004 aimed to compare the nutritional status of 25 people with HD versus 25 control subjects by analyzing both anthropometric and biochemical indicators, energy and macronutrient intake in order to identify which of above mentioned indicators was altered in people with HD. The anthropometric indicators such as weight (p=0.0047), BMI (p= 0.0001) and body fat (p= 0.00004) were lower in the HD group compared to the control group, thus certain anthropometric indicators were altered in people with HD. The biochemical indicators of albumin, haemoglobin, haematocrit, total lymphocyte count, glucose, cholesterol and triacylglycerol (p=0.637, p=0.536, p=0.186, p=0.668, p=0.759, p=0.116 and p=0.605, respectively), were reduced in the HD group but they did not reach statistical significance. This indicates their possible alteration in people with HD compared to controls. Finally, dietary intake was assessed and demonstrated that mean energy intake was significantly higher in people with HD compared to controls (p=0.003), most likely due to the higher consumption of carbohydrates (p=0.003).

A study by Rivadeneyra *et al*., 2016 analysed and investigated factors associated with MD adherence and dietary intake in HD. The study consisted of ninety-eight Spanish people with HD and pre-manifesting HD carriers of the EHDN. The study found that participants with moderate or high MD adherence were characterized by having higher intake of cereals (p=0.041), alcohol (p<0.001), fish (p=0.004) and MUFA/SFA (P <0.001) and lower consumption of dairy products (p=0.02) compared to participants with low MD adherence. Furthermore, moderate MD adherence was associated with older age (p=0.031), decreased comorbidity (p=0.018), and low motor UHDRS score (p=0.041) compared low MD adherence whereas high MD adherence was associated with lower UHDRS motor scores (p=0.033) compared to moderate MD adherence. Dietary factors such as fiber (p=0.029), PUFA/SFA (p=0.010), PUFA + MUFA/SFA (p<0.001), MUFA/SFA (p<0.001), biotin (p=0.002), folic acid (p=0.030), vitamin C (p=0.024), vitamin E (p=0.008), copper (p=0.016) and selenium (p=0.001) were observed to be increased in participants with moderate or high MD adherence. MUFA/SFA intake showed a slight correlation with better TFC and UHDRS cognitive score. Factors such as better quality of life (p=0.009), reduced comorbidity, lower UHDRS motor scores and low psychiatric comorbidity were associated with participants with moderate/high MD adherence. However, overall HD severity was similar between participants of low vs moderate/high MD adherence.

**High protein diet and HD**

A study by Chen *et al*., 2015 investigated the effects of a high protein diet on HD. The study assessed the possible impact of high dietary protein in 30 people with HD. Then 14 people with HD out of the 30 were followed up for a 2-year period pilot study and 22 people with HD were followed up for a 1-year period study. The 30 people with HD were recruited for a 12-day hospitalization. A standard protein diet (SPD) was given for 5 days and then blood samples were collected to measure arterial ammonia levels and venous citrulline levels. Afterwards, a high protein diet (HPD) was given for another 5 days, and blood samples were collected on day 12 to measure arterial ammonia levels and venous citrulline levels. The HPD caused a slight increase in venous blood citrulline levels (p=0.0378), but blood ammonia levels (p=0.9791) were not affected by HPD. The 2-year pilot follow-up study was conducted in order to determine if blood citrulline concentration can be used as a biomarker for HD progression. A blood sample was collected every 6 months from 14 people with HD where UHDRS and MMSE were assessed. Blood citrulline levels were higher in people with HD compared to non-HD controls, but blood citrulline concentration did not change significantly in people with HD during the 2 year follow up. Furthermore, blood citrulline levels did not show significant correlation with UHDRS score (p= 0.0985), IS (p=0.2144) and functional capacity (p=0.5010). Finally, an association between disease duration and blood citrulline levels was also investigated but there was a negative correlation with disease duration and blood citrulline levels (p=0.0221).
